# Neutrophil extracellular trap-microparticle complexes enhance thrombin generation via the intrinsic pathway of coagulation in mice

**DOI:** 10.1038/s41598-018-22156-5

**Published:** 2018-03-05

**Authors:** Yongzhi Wang, Lingtao Luo, Oscar Ö Braun, Johannes Westman, Raed Madhi, Heiko Herwald, Matthias Mörgelin, Henrik Thorlacius

**Affiliations:** 1 Department of Clinical Sciences, Malmö, Section for Surgery, Lund University, Lund, Sweden; 2 Department of Clinical Sciences, Lund, Section of Cardiology, Lund University, Lund, Sweden; 3 Department of Clinical Sciences, Lund, Division of Infection Medicine, Lund University, Lund, Sweden; 4 Department of Surgery, The First Affiliated Hospital of Xiamen University, Xiamen, China; 5 Program in Cell Biology, The Hospital for Sick Children, Toronto, Canada

## Abstract

Abdominal sepsis is associated with dysfunctional hemostasis. Thrombin generation (TG) is a rate-limiting step in systemic coagulation. Neutrophils can expell neutrophil extracellular traps (NETs) and/or microparticles (MPs) although their role in pathological coagulation remains elusive. Cecal ligation and puncture (CLP)-induced TG *in vivo* was reflected by a reduced capacity of plasma from septic animals to generate thrombin. Depletion of neutrophils increased TG in plasma from CLP mice. Sepsis was associated with increased histone 3 citrullination in neutrophils and plasma levels of cell-free DNA and DNA-histone complexes and administration of DNAse not only eliminated NET formation but also elevated TG in sepsis. Isolated NETs increased TG and co-incubation with DNAse abolished NET-induced formation of thrombin. TG triggered by NETs was inhibited by blocking factor XII and abolished in factor XII-deficient plasma but intact in factor VII-deficient plasma. Activation of neutrophils simultaneously generated large amount of neutrophil-derived MPs, which were found to bind to NETs via histone-phosphatidylserine interactions. These findings show for the first time that NETs and MPs physically interact, and that NETs might constitute a functional assembly platform for MPs. We conclude that NET-MP complexes induce TG via the intrinsic pathway of coagulation and that neutrophil-derived MPs play a key role in NET-dependent coagulation.

## Introduction

Abdominal sepsis is a leading cause of mortality in intensive care units^1^. Sepsis-induced mortality rate ranges generally between 18–30% and increases further in the presence of disseminated intravascular coagulation^2,3^. The host response in sepsis is characterized by wide-spread activation of innate immune cells and alterations in the coagulation system^4^. It is well-accepted that neutrophils play a central role in sepsis^5^. On one hand, neutrophils are necessary for eliminating invading microbes but on the other hand, excessive neutrophil activation causes tissue damage and organ failure^6,7^. Dysfunctional coagulation is one of the most prominent features in sepsis, typified by an early hypercoagulable phase concomitant with impaired anticoagulation and fibrinolysis^8^ followed by a hypocoagulable phase due to consumption of platelets and coagulation factors^9^. Convincing evidence has documented that thrombin generation (TG) constitutes a rate-limiting step in the coagulation cascade by cleaving fibrinogen into fibrin^10^. Exaggerated pulmonary deposition of fibrin deposition reduces lung compliance and jeopardizes gaseous exchange in sepsis^11^. Thrombin is generated by proteolytic cleavage of prothrombin, which is secreted from hepatocytes^12^. Moreover, thrombin is a potent pro-inflammatory mediator with the capacity to activate monocytes and endothelial cells via specific protease-activated receptors^13^. Thus, thrombin formation might be a critical link between coagulation and inflammation in sepsis. Hemostatic cascades are complex processes consisting of a dynamic interplay between several discrete elements^4^. Global hemostasis assays, including TG tests have emerged as effective tools to obtain more comprehensive evaluations of hemostasis^9^. For example, TG has been shown to be useful in the evaluation of diseases, such as chronic liver disease^14^, trauma-induced coagulopathy^15^ and organ transplantation^16^. Nonetheless, the role of neutrophils in sepsis-induced thrombin formation and dysfunctional coagulation remains elusive.

Activated neutrophils undergo several changes of importance for the host defense against infections. The primary responses of neutrophils exposed to invading microbes are the formation of reactive oxygen species and engulfment of pathogens into phagosomes^17^. In addition, recent findings have shown that activated neutrophils can also expel nuclear DNA to form web-like structures containing nuclear, cytoplasmatic and granular proteins, which referred to as neutrophil extracellular traps (NETs)^18^. Convincing data have shown that protein arginine deiminase 4 (PAD4) is critical for NET formation although more research is needed to understand signaling pathways regulating expulsion of DNA from neutrophils^19^. NETs have been shown to exhibit antimicrobial functions by trapping and killing bacterial and fungal invaders^18,20^. In addition, exaggerated NET formation has been demonstrated to cause tissue damage in models of inflammation and infection^21^. In this context, it is interesting to note that compelling evidence has shown that NETs can promote thrombosis^22^ and coagulation^23^. However, the impact and mechanisms of NETs on TG are partially understood. Activated neutrophils also shed off microparticles (MPs). MPs are sphere-shaped intact vesicles released from cell membranes with a size less than 1 μm^24^. In contrast to their quiescent mother cells, MPs contain large amounts of phophatidylserine (PS) on their outer surface^25^. PS is negatively charged and is therefore able to attract procoagulant factors^26^. However, the role of neutrophil-derived MPs in sepsis-evoked TG is not known.

Based on the considerations above, we hypothesized herein that neutrophils might play an important role in systemic coagulation via formation of NETs and/or MPs in abdominal sepsis. For this purpose, we used a sepsis model based on ligation and puncture of the cecum.

## Results

### Neutrophils regulate systemic inflammation in abdominal sepsis

Administration of the anti-Ly6G antibody decreased circulating numbers of neutrophils by more than 97% (Supplemental Fig. 1) while having no concomitant effect on the numbers of monocytes, T-cells or B-cells in the circulation (Supplemental Fig. 1). CLP increased MPO levels in the lung by 5.6-fold (Supplemental Fig. 2A). Depletion of neutrophils decreased CLP-induced lung MPO activity by 86% (Supplemental Fig. 2A). Moreover, CLP markedly increased pulmonary levels of CXCL1 (Supplemental Fig. 2B) as well as plasma levels of IL-6 (Supplemental Fig. 2C) and CXCL1 (Supplemental Fig. 2D). In addition, neutrophil depletion attenuated CLP-induced pulmonary levels of CXCL1 by 40% (Supplemental Fig. 2B). Depletion of neutrophils reduced plasma levels of IL-6 by 69% (Supplemental Fig. 2C) and CXCL1 by 61% (Supplemental Fig. 2D) in septic animals. These findings suggest that neutrophils play an important role in the systemic inflammatory response in abdominal sepsis.

### Neutrophils regulate TG in abdominal sepsis

TAT complexes are formed following neutralization of thrombin by anti-thrombin III and have been used as a surrogate marker for TG^27^. Herein, we found that CLP markedly increased plasma levels of TAT complexes (Supplemental Fig. 3A) supporting the notion that CLP elevates TG in mice. This increase in thrombin formation in sepsis *in vivo* is reflected by a decreased capacity of plasma from septic animals in generating thrombin *ex vivo* (Supplemental Fig. 3B), which is related to a consumption of coagulation factors and thrombin in the septic animals^3^. Indeed, we observed that peak thrombin formation was reduced by 45% (Supplemental Fig. 3C) and total TG was decreased by 46% (Supplemental Fig. 3D) in plasma from septic mice. Notably, neutrophil depletion restored peak and total TG in plasma from CLP mice to similar levels as those in sham animals (Supplemental Fig. 3B–D), indicating that neutrophils play a key role in sepsis-induced thrombin formation. This notion is in line with the observation that neutrophil depletion reduced plasma levels of TAT in septic animals (Supplemental Fig. 3A).

### NET-dependent generation of thrombin

**Figure 1:**
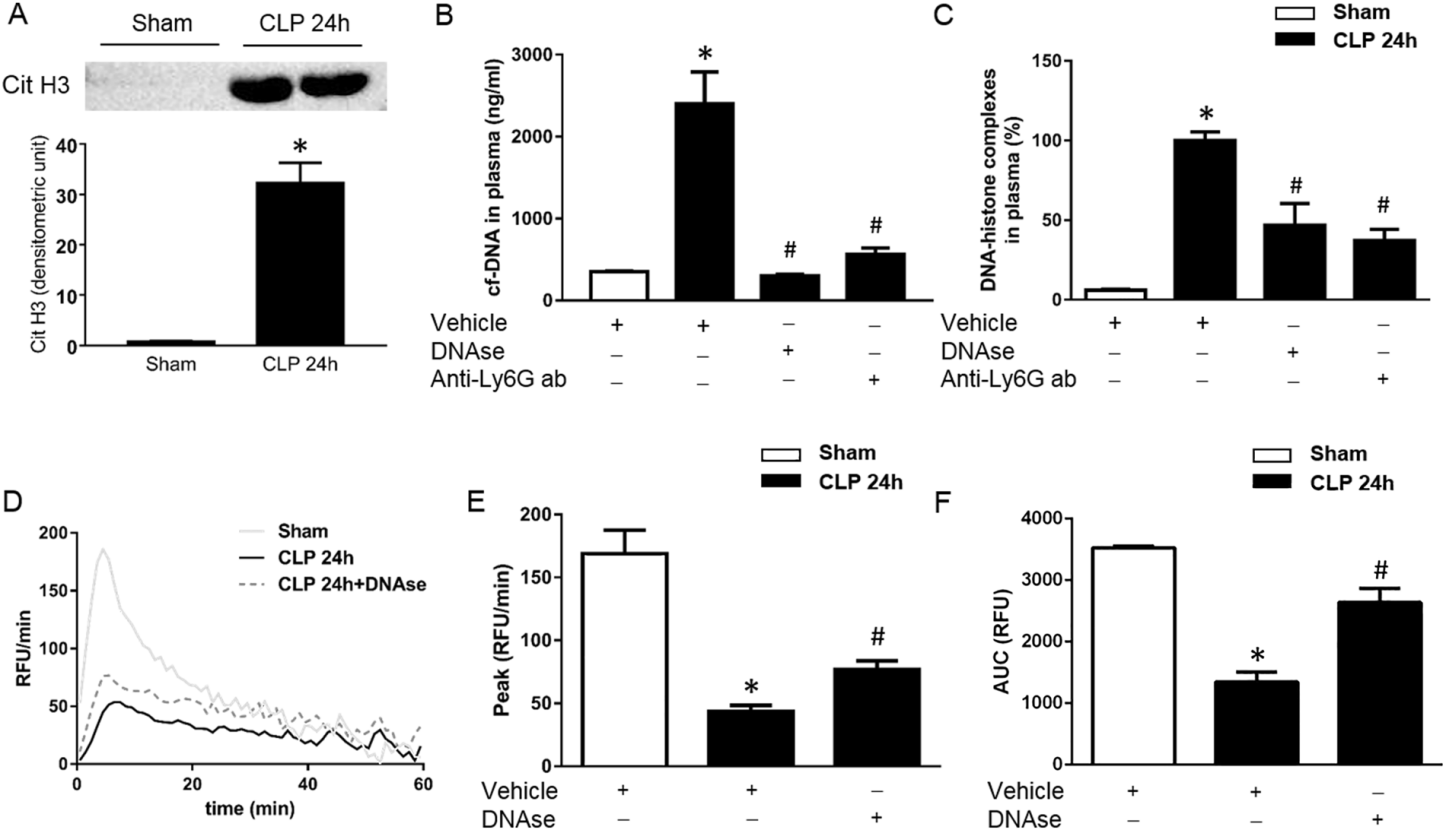
NET formation in abdominal sepsis. (**A**) Representative western blot and aggregate data on citrullinated histone 3 in neutrophils isolated from the blood from sham and CLP animals. Plasma levels of (**B**) cf-DNA and (**C**) DNA-histone complexes were determined 24 h after CLP. (**D**) TG over time, (**E**) peak and (**F**) total levels of TG were determined as described in Material and Methods. Mice underwent CLP or the identical laparotomy and resuscitation procedures, but the cecum was neither ligated nor punctured (Sham). Animals received intraperitoneal injections of DNAse (5 mg/kg), an anti-Ly-6G antibody (Anti-Ly-6G ab, 20 mg/kg) or vehicle. Data are presented as mean ± SEM and *n* = 5. *P < 0.05 *vs*. Sham and ^#^*P* < 0.05 *vs*. Vehicle + CLP.

CLP increased levels of citrullinated histone 3 in neutrophils isolated from blood of septic mice (Fig. 1A) and plasma levels of cf-DNA by nearly six-fold (Fig. 1B) as well as DNA-histone complexes by more than 15-fold (Fig. 1C), suggesting that CLP is associated with increased NET formation in the circulation. This notion is also supported by separate experiments showing co-localization of citrullinated histone 3 and MPO on blood neutrophils from CLP animals (Supplemental Fig. 4). Notably, administration of DNAse decreased plasma levels of cf-DNA by 87% and DNA-histone complexes by 42% in septic animals (Fig. 1B–C), indicating that DNAse effectively reduces NET levels in septic animals. We next examined the effect of DNAse on sepsis-induced TG. Typical curves of TG from the different groups of animals were depicted in Fig. 1D. We found that DNAse increased peak formation by 90% (Fig. 1E) and total TG by 96% (Fig. 1F) in plasma from septic mice, indicating that degradation of NETs markedly antagonized sepsis-induced thrombin formation. This is supported by the reduction of TAT levels in the plasma of septic animals treated with DNAse (Supplemental Fig. 3A).

### NET and MP interactions regulate TG

**Figure 2:**
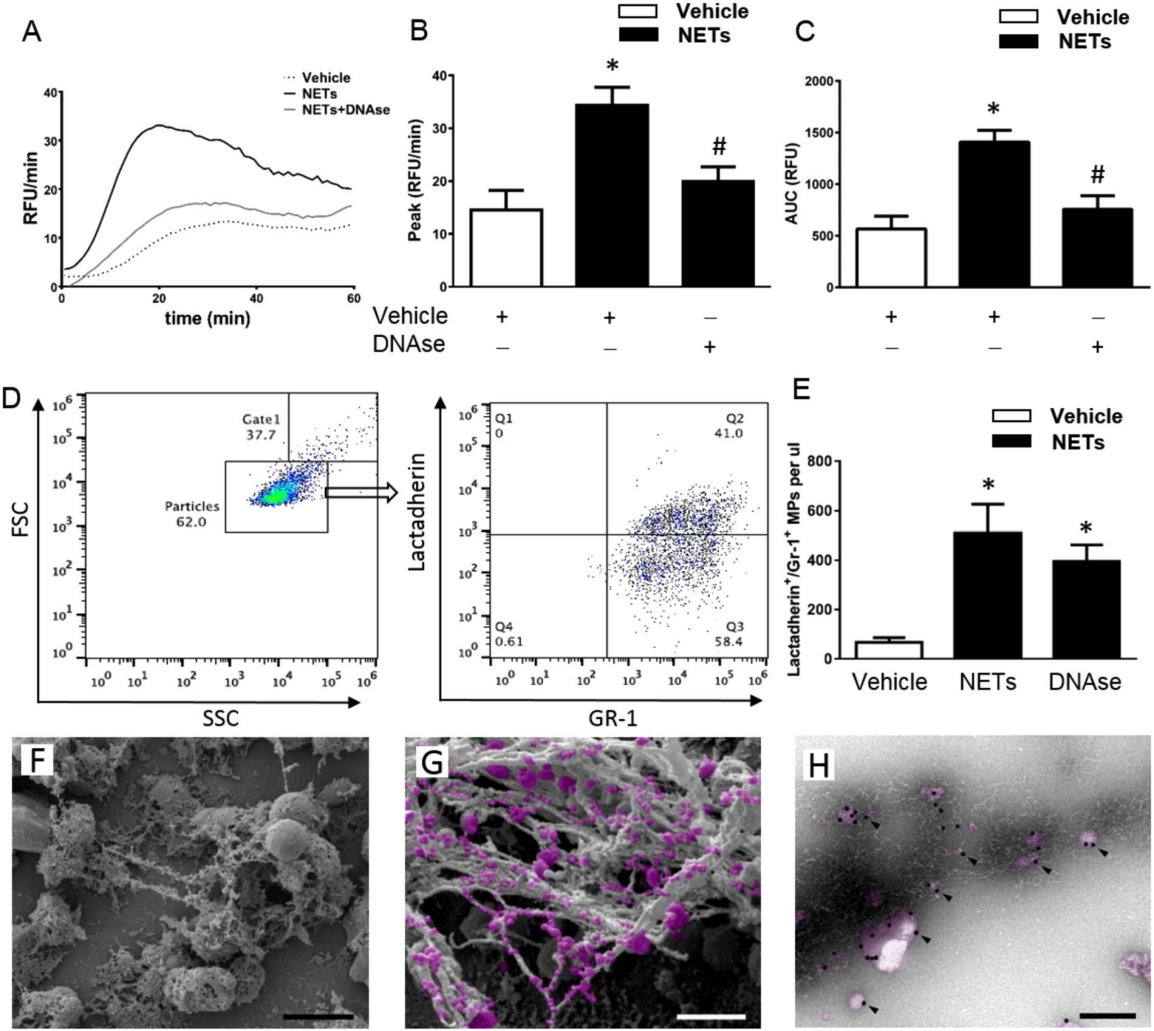
NET-induced generation of thrombin. NETs were generated from PMA-stimulated bone marrow neutrophils. PPP was co-incubated with NETs containing vehicle or DNAse. (**A**) TG over time, (**B**) peak and (**C**) total levels of TG were determined as described in Material and Methods. Supernatants from PMA-stimulated neutrophils were incubated with FITC-conjugated bovine lactadherin and a PerCP Cy5.5-conjugated anti-Gr-1 antibody. (**D**) Particles were gated as events smaller than 1 μm in size and then gated as Lactadherin^+^/Gr-1^+^ and quantified by use of flow cytometry. (**E**) Aggregate data on NET-induced MPs. (**F**) Scanning electron microscopy showing neutrophils with extracellular web-like structures. Scale bar = 5 μm. (**G**) A higher magnification showing MPs attached to neutrophil-derived NETs. Scale bar = 2 μm. MPs are denoted in pink color. H) Transmission electron microscopy showing NETs and MPs incubated with gold-labeled annexin V (black particles, black arrowhead). Scale bar = 0.25 µm. Data are presented as mean ± SEM and *n* = 5. *P < 0.05 *vs*. Vehicle and ^#^*P* < 0.05 *vs*. Vehicle + NETs.

**Figure 3:**
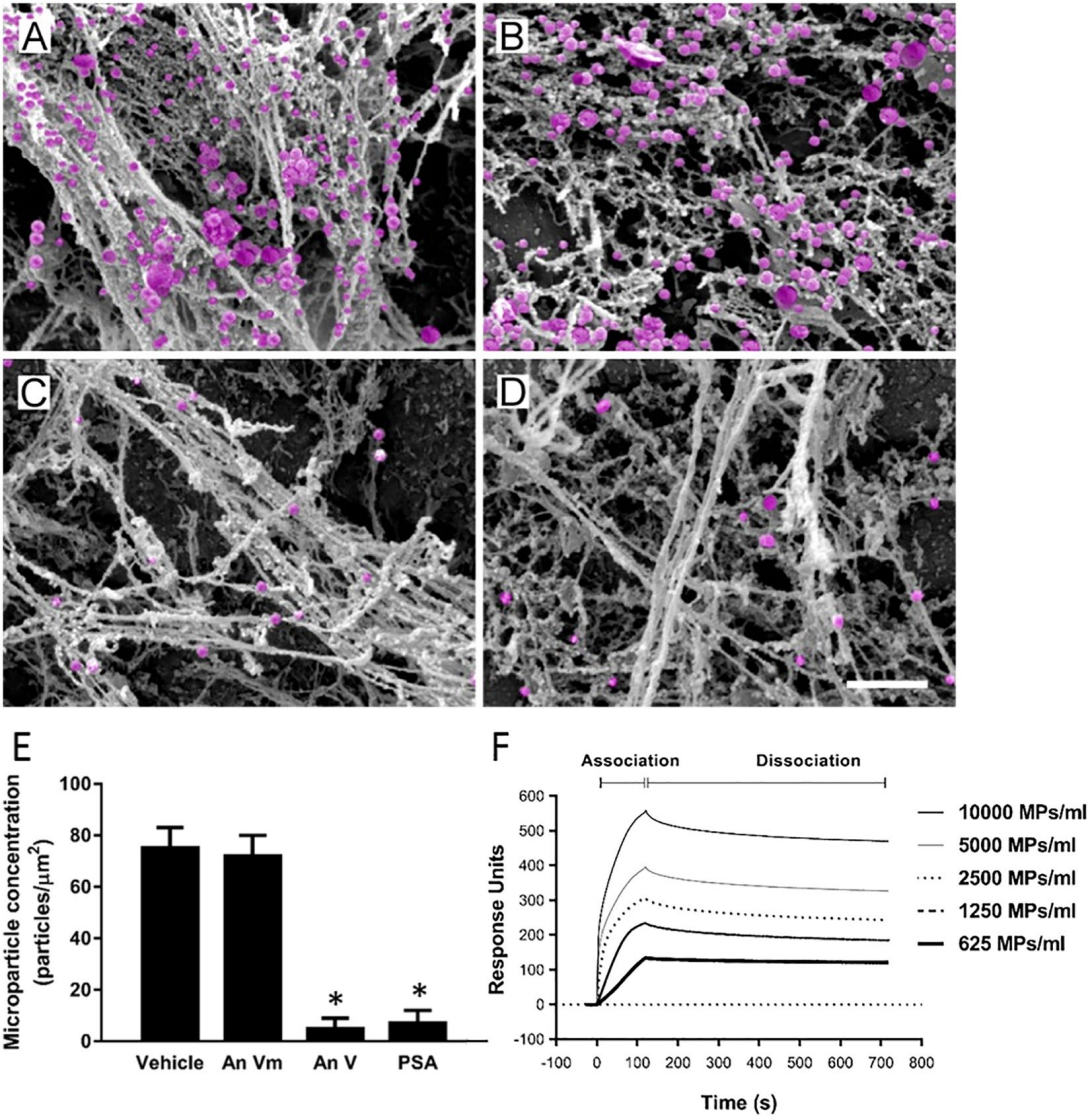
MP binding to NETs. NETs were generated from PMA-stimulated bone marrow neutrophils co-incubated with (**A**) vehicle (**B**) mutated annexin V (An Vm), (**C**) wild-type annexin V (An V) or (**D**) polysialic acid (PSA). Scanning electron microscopy showing MPs attached to neutrophil-derived NETs. Scale bar = 2 μm. (**E**) Aggregate data on MP binding to NETs. (**F**) Surface plasmon resonance with histone H4 immobilized to a CM5 sensorchip. Indicated concentrations of MPs were applied in a flow over the sensorchip surface as described in Methods. Data are presented as mean ± SEM and *n* = 5. *P < 0.05 *vs*. Vehicle + CLP.

NETs were generated by stimulating mouse bone marrow neutrophils with PMA and contained Mac-1, elastase and MPO but not CD41 (Supplemental Fig. 5), supporting the notion that these structures are NETs and that there were no signs of platelet contamination of the samples. Isolated NETs effectively increased TG *ex vivo* (Fig. 2A). For example, NETs increased peak formation of thrombin by 2.4-fold (Fig. 2B) and enhanced total TG by 2.5-fold (Fig. 2C). Co-incubation with DNAse abolished the effects of NETs on thrombin formation (Fig. 2A–C). In parallel to NET formation, activated neutrophils also shed off membrane-derived MPs^24^ and we therefore asked whether neutrophil-derived MPs could be involved in NET-dependent TG. We first observed that PMA stimulation of neutrophils not only triggered formation of NETs but also generated large amounts of MPs (Fig. 2D). Quantification revealed that PMA challenge increased MP formation by more than 7-fold (Fig. 2E). Notably, co-incubation with DNAse had no effect on the number of neutrophil-derived MPs formed in response to PMA stimulation (Fig. 2E). By use of scanning electron microscopy, it was found that PMA challenge induced formation of neutrophil extracellular fibrillar web-like structures (Fig. 2F) coated with multiple MPs (Fig. 2G). Transmission immunoelectron microscopy revealed that MPs associated with the NETs bound annexin V but annexin V did not bind directly to NETs (Fig. 2H), demonstrating that NETs constitute an assembly platform for MPs. We next wanted to define the molecular interaction between NETs and MPs. For this purpose, we used a mutated annexin (M1234), which lacks the ability to bind to PS and a recombinant wild-type annexin V^28^. Again it was observed that NETs formation was associated with widespread binding of MPs (Fig. 3A). Co-incubation with mutated annexin V (M1234-Annexin V) as a negative control had no effect on interactions between NETs and MPs (Fig. 3B). In contrast, co-incubation with wild-type annexin V abolished MP binding to NETs (Fig. 3C). Polysialic acid (PSA) is a highly negatively charged glycan that directly binds to histones^29^ and has been demonstrated to block NET- and histone-induced cell cytotoxicity^30^. Herein, we found that co-incubation with PSA abolished MP binding to NETs (Fig. 3D). Quantification revealed that wild-type annexin V and PSA reduced MP binding to NETs by 93% and by 91%, respectively (Fig. 3E). In separate experiments, we used surface plasmon resonance technology to determine the binding of neutrophil-derived MPs to histones. Histone H4 was immobilized to a CM5 sensorchip and different concentrations of MPs were applied in a flow over the surface. Data show that neutrophil-derived MPs dose-dependently bound to histone H4 (Fig. 3F).

### Functional role of NET-MP complexes in coagulation

**Figure 4:**
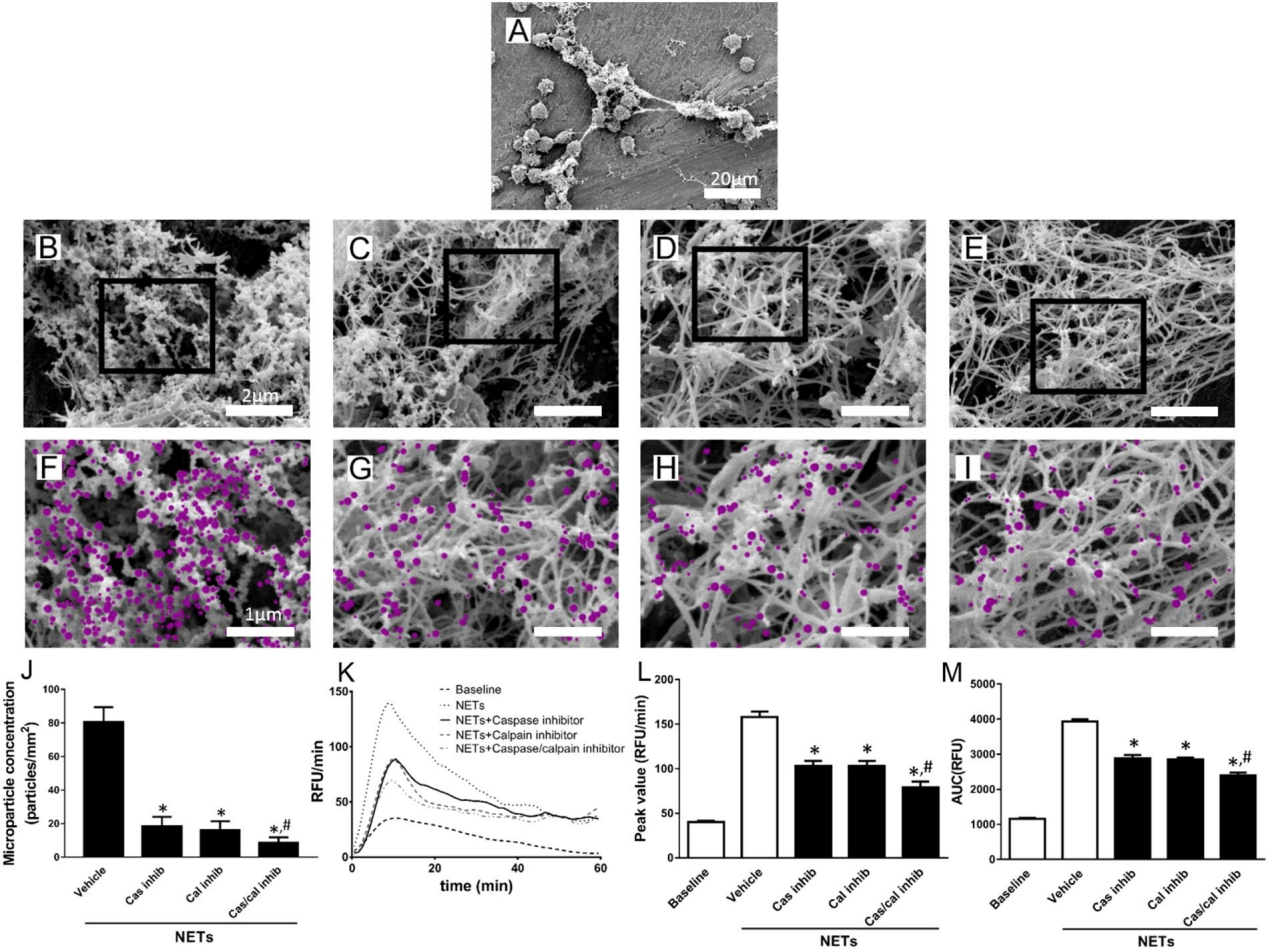
Functional role of NET-MP complexes. NETs were generated from PMA-stimulated bone marrow neutrophils (**A**). Scale bar = 20 µm. Then co-incubated with (**B**,**F**) vehicle (**C**,**G**) caspase inhibitor, (**D**,**H**) calpain inhibitor or (**E**,**I**) a combination of caspase and calpain inhibitors. Scanning electron microscopy showing MPs attached to neutrophil-derived NETs. Scale bar = 2 μm in the top insert and a higher magnification of the indicated area of interest from the top insert is shown below with a scale bar = 1 μm. MPs are denoted in pink color in the lower inserts. (**J**) Aggregate data on MP binding to NETs. (**K**) TG over time, (**L**) peak and (**M**) total levels of TG were determined as described in Material and Methods. Data are presented as mean ± SEM and *n* = 5. *P < 0.05 *vs*. Vehicle and ^#^*P* < 0.05 *vs*. Vehicle + NETs.

**Figure 5:**
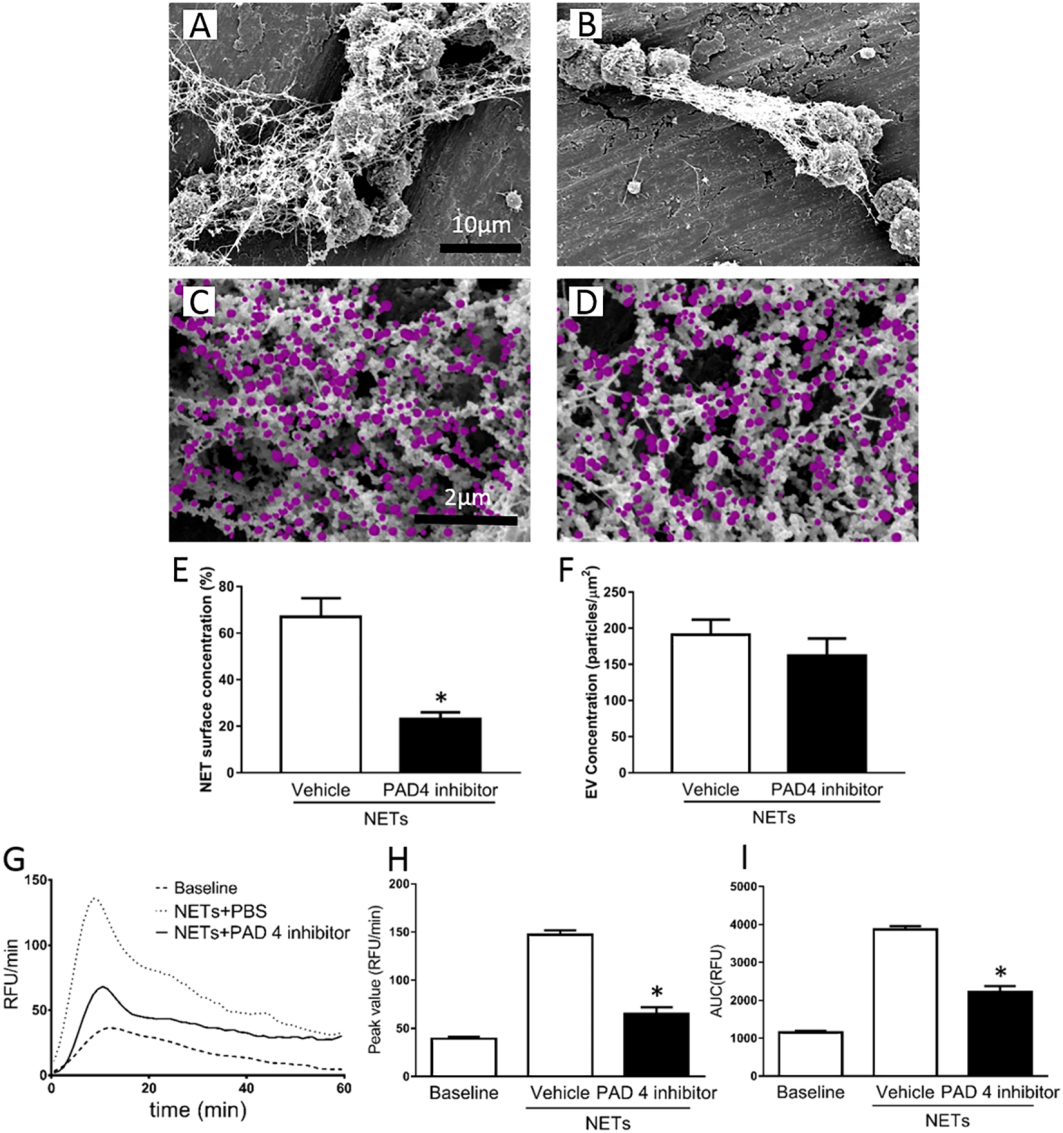
Role of PAD4 in formation of NET-MP complexes. NETs were generated from PMA-stimulated bone marrow neutrophils co-incubated with (**A**,**C**) vehicle or (**B**,**D**) Cl-amidine, a PAD4 inhibitor. (**A**,**B**) Scanning electron microscopy showing MPs attached to neutrophil-derived NETs. Scale bar = 10μm and (**C**,**D**) a higher magnification with a scale bar = 2 μm. (**C**,**D**) MPs are denoted in pink color. Aggregate data on (**E**) NET formation and (**F**) MP per μm^2^ of of NETs. (**G**) TG over time, (**H**) peak and (**I**) total levels of TG were determined as described in Material and Methods. Data are presented as mean ± SEM and *n* = 5. *P < 0.05 *vs*. Vehicle + NETs.

The number of MPs per surface area of NETs was studied in PMA-stimulated neutrophils (Fig. 4A). Widespread binding of MPs to NETs were found around PMA-stimulated neutrophils treated with vehicle (Fig. 4B,F). In contrast, co-incubation of PMA-stimulated neutrophils with a caspase (Fig. 4C,G) and a calpain (Fig. 4D,H) inhibitor or the combination (Fig. 4E,I) markedly decreased MP binding to NETs. Quantification revealed that inhibition of caspase and calpain reduced MP binding to NETs by 77% and 80%, respectively (Fig. 4J). Combined inhibition of caspase and calpain decreased the number of MP on NETs by 89% (Fig. 4J). Interestingly, inhibition of caspase and/or calpain had no effect on the DNA (Supplemental Fig. 6A) or histone H4 (Supplemental Fig. 6B) content in NETs expelled by PMA-stimulated neutrophils. Notably, we found that NETs depleted of MPs by co-incubation with inhibitors of caspase and/or calpain had a significantly lower capacity to trigger TG (Fig. 4K). For example, co-incubation with caspase and calpain inhibitors decreased NET-induced peak formation of thrombin by 50% (Fig. 4L) and total TG by 39% (Fig. 4M). As expected Cl-amidine, a PAD4 inhibitor, markedly reduced PMA-induced NET formation (Fig. 5A–F). Interestingly, we found that inhibition of PAD4 had no effect on MP formation and binding to NETs (Fig. 5C–F). Moreover, it was found that NETs generated in the presence of Cl-amidine decreased thrombin generation (Fig. 5G). For example, the PAD4 inhibitor reduced NET-induced peak and total formation of thrombin by 56% and 43%, respectively (Fig. 5H–I).

### NET-MP complexes increase TG via the intrinsic pathway of coagulation

**Figure 6:**
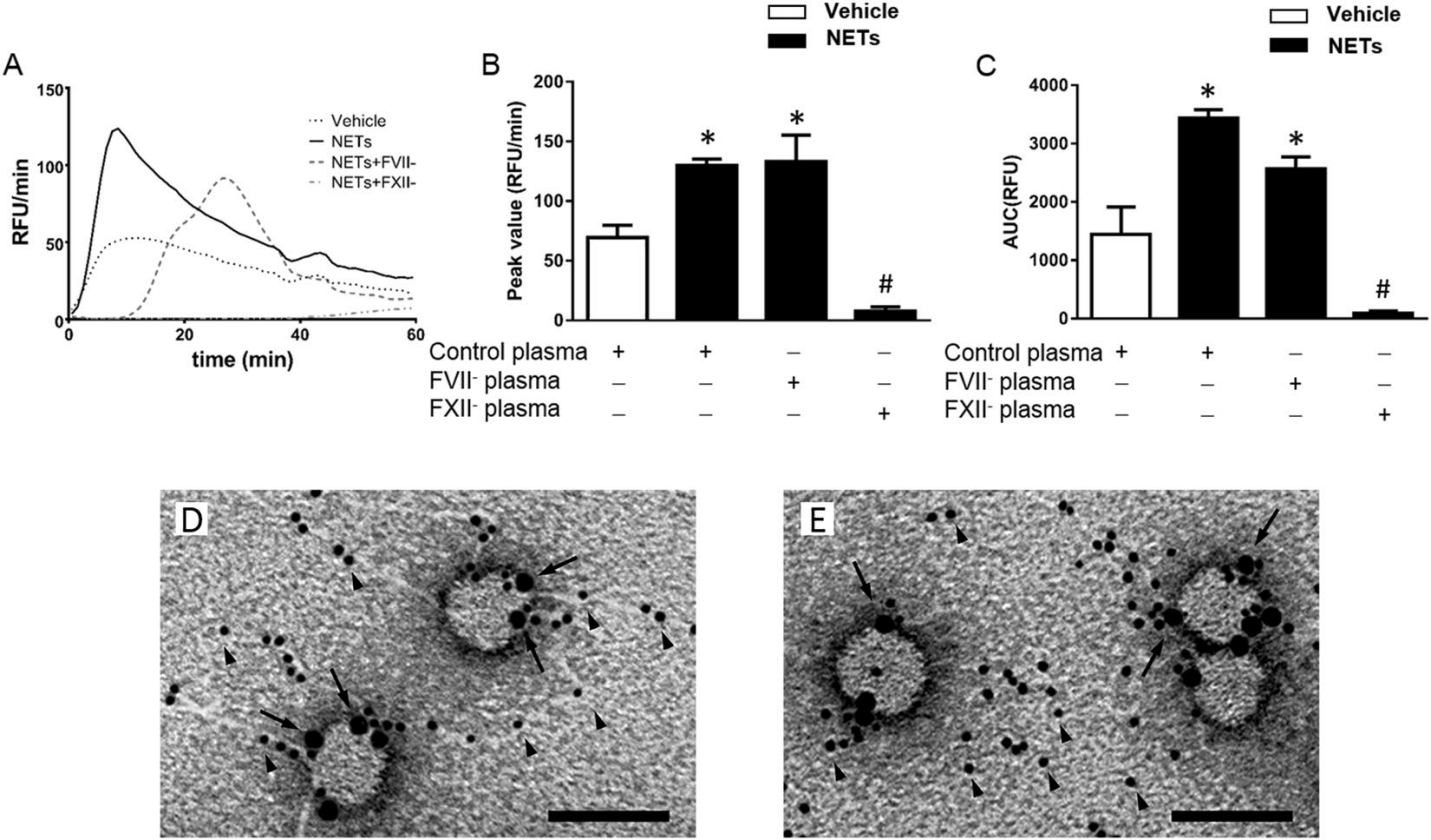
NETs regulate TG via the intrinsic pathway of coagulation. NETs generated from PMA-stimulated bone marrow neutrophils were incubated in wild-type, factor VII- (FVII^−^ plasma) and factor XII-deficient (FXII^−^ plasma) PPP. (**A**) TG over time, (**B**) peak and (**C**) total levels of TG were determined as described in Material and Methods. Transmission electron microscopy showing NETs and MPs incubated with a gold-labeled anti-histone H4 antibody (small gold particles, black arrowhead) and (**D**) gold-labeled annexin V (large gold particles, black arrow) or (**E**) a gold-labeled anti-FXII antibody (large gold particles, black arrow). Scale bar = 0.25 µm. Data are presented as mean ± SEM and *n* = 5. *P < 0.05 *vs*. Vehicle + Control plasma and ^#^*P* < 0.05 *vs*. NETs + Control plasma.

Incubation of NETs in Factor VII-deficient plasma had no effect on NET-provoked generation of thrombin (Fig. 6A–C). In contrast, incubation of NETs in Factor XII-deficient plasma reduced NET-induced peak formation by 94% and total TG by 97% (Fig. 6B–C). In addition, we co-incubated NETs with corn trypsin inhibitor (CTI), a specific inhibitor of factor XIIa, and found that CTI decreased peak formation by 91% and total TG by 89% triggered by NET-MP complexes (Supplemental Fig. 7A–C). By use of transmission immunoelectron microscopy we found that MPs associated with NETs not only bound to annexin V (Fig. 6D) but also to Factor XII (Fig. 6E).

### NETs interact with MPs *in vivo*

**Figure 7:**
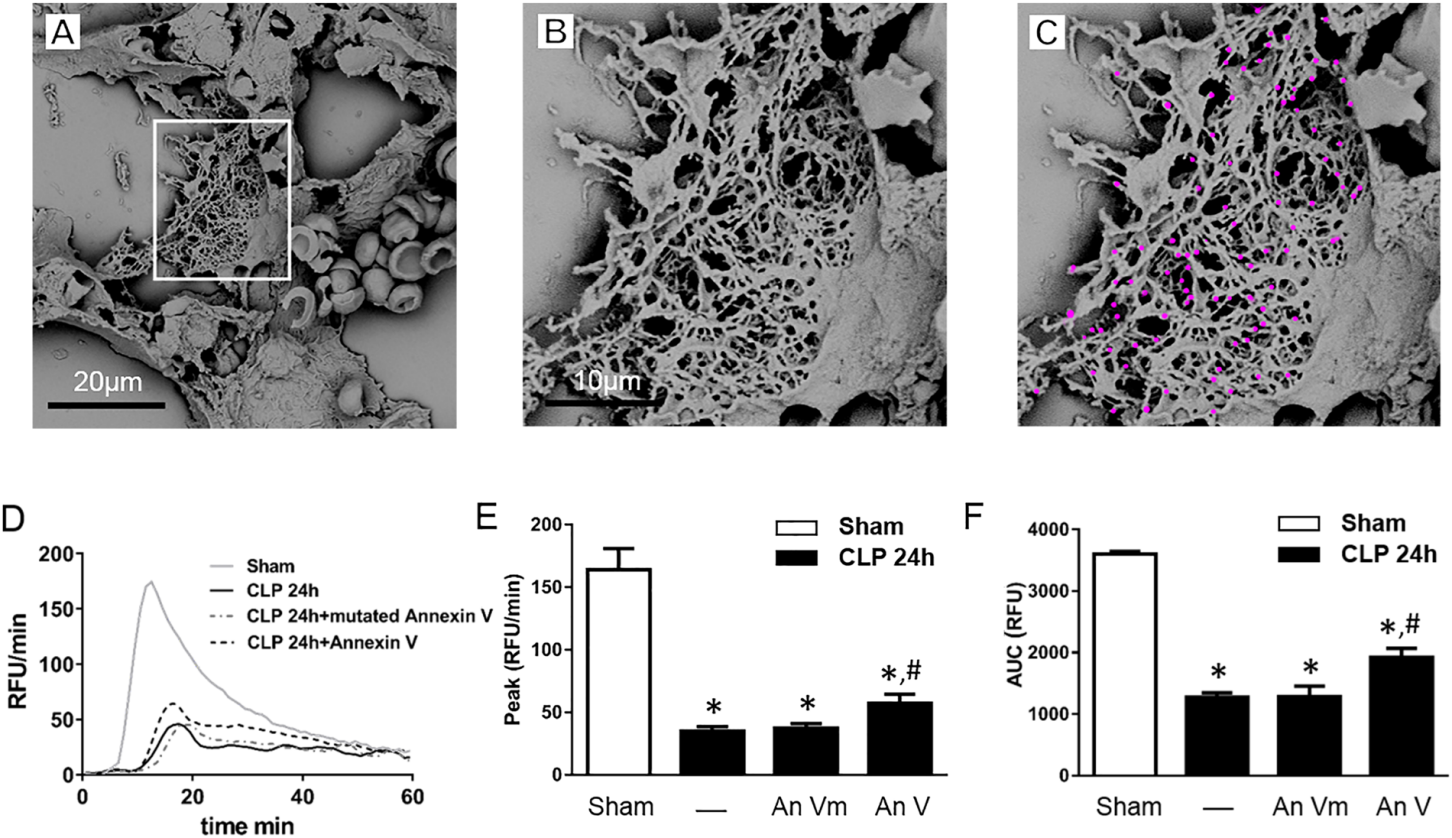
Sepsis-induced formation of NET-MP complexes. (**A**) Scanning electron microscopy showing extracellular web-like structures in the lung from a mouse exposed to CLP. Scale bar = 20 μm. (**B**) A higher magnification of the indicated area of interest from Figure A showing MPs attached to NETs in the septic lung. (**C**) MPs are denoted in pink color. Scale bar = 10 μm. (**D**) TG over time, (**E**) peak and (**F**) total levels of TG were determined as described in Material and Methods. Mice underwent CLP or the identical laparotomy and resuscitation procedures, but the cecum was neither ligated nor punctured (Sham). Animals received intraperitoneal injections of wild-type annexin V (An V) or mutated annexin V (An Vm). Data are presented as mean ± SEM and *n* = 5. *P < 0.05 *vs*. Sham and ^#^*P* < 0.05 *vs*. An Vm + CLP.

By use of scanning electron microscopy, we found that CLP triggered formation of extracellular fibrillar and web-like structures in the inflamed lung compatible with NETs (Fig. 7A–C), which were not observed in the normal lung (not shown). Moreover, by applying scanning electron microscopy, we found that CLP-induced NETs in the lung contained numerous round structures compatible with MPs (Fig. 7C). Considering our finding that annexin V blocks NET and MP interactions, we wanted to examine the effect of blocking these interactions on TG *in vivo* (Fig. 7D). Notably, treatment with wild-type annexin V increased peak (Fig. 7E) and total (Fig. 7F) TG in plasma form septic animals as compared with septic animals receiving mutated annexin V (M1234-Annexin V).

### Formation of NET-MP complexes in human neutrophils

**Figure 8:**
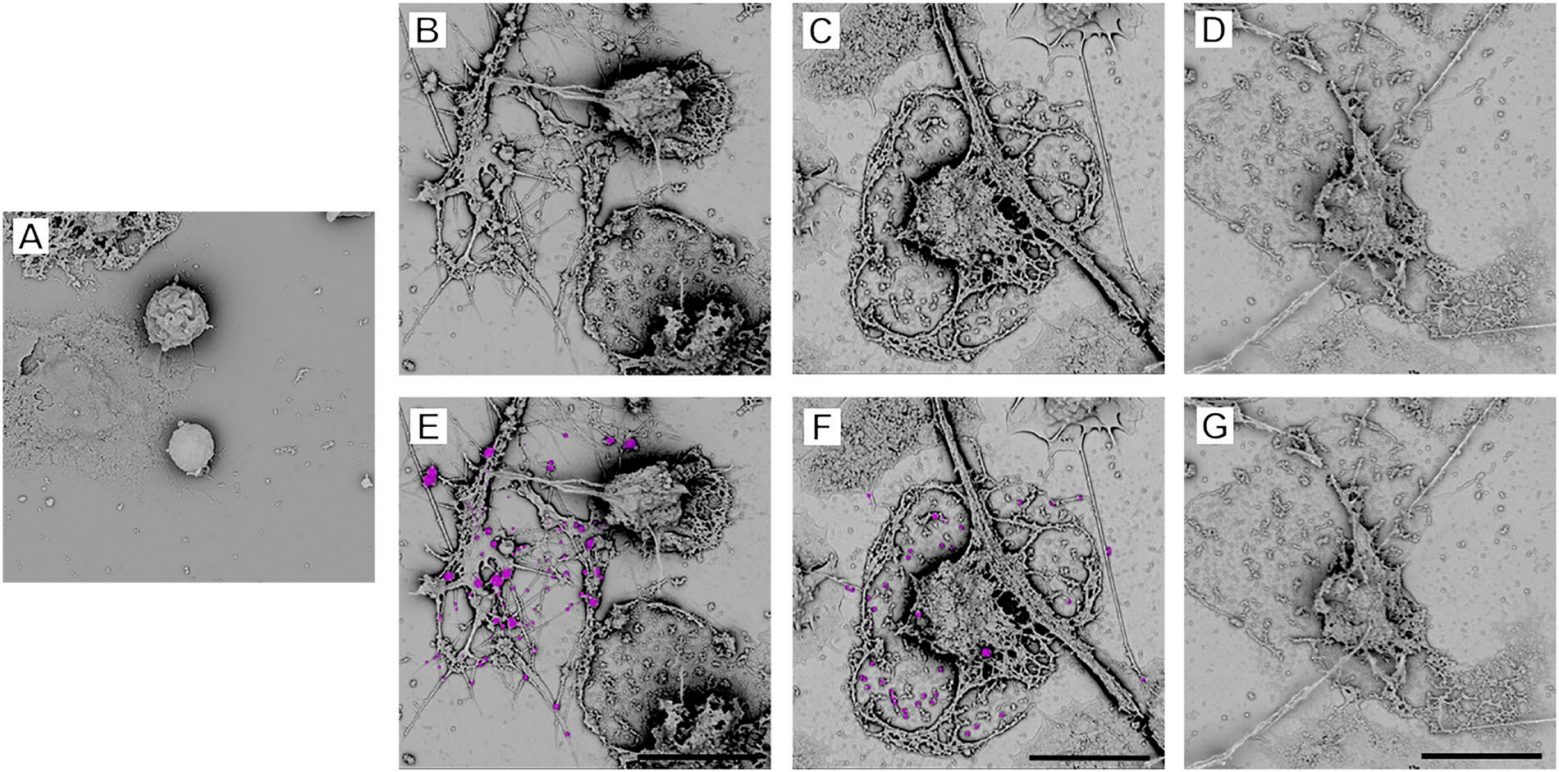
NET-MP complex formation in human neutrophils. (**A**) Resting human neutrophils. NETs were generated from M1 protein-stimulated (2 h) human neutrophils co-incubated with (**B**,**E**) vehicle, (**C**,**F**) mutated annexin V and (**D**,**G**) wild-type annexin V. Scanning electron microscopy showing MPs attached to neutrophil-derived NETs. MPs are denoted in pink color in the lower inserts. Scale bar = 10 μm. One representative experiment of 5 independent experiments is shown.

Our findings indicated that NETs and MPs form aggregates and may therefore represent a potential therapeutic target. Therefore, we next examined the clinical relevance of these experimental findings in human neutrophils stimulated with M1 protein. The M1 serotype of *Streptococcus pyogenes* is widely recognized as the most frequent serotype linked with fatal streptococcal toxic shock syndrome^31^. It was found that M1 protein was a potent inducer of NET formation in human neutrophils (Fig. 8A–G). Notably, NETs triggered by M1 protein exhibited wide-spread binding of MPs. Moreover, co-incubation with wild-type annexin V markedly decreased NET-MP complex formation (Fig. 8D,G) whereas co-incubation with mutated annexin V (M1234) had no effect on MP binding to NETs expelled by human neutrophils stimulated with M1 protein (Fig. 8C,F).

## Discussion

Significant alterations in the circulating blood are a hallmark of the systemic response to bacterial infections. We show that neutrophils not only play an important role in the systemic inflammatory response but also in the coagulative changes in sepsis through formation of NETs. Our results demonstrate that NETs promote formation of thrombin through the intrinsic pathway of coagulation. Moreover, we found that NETs via histone-PS interactions serve as an assembly platform for MPs. These findings delineate novel mechanisms regulating TG and might pave the way for new and specific targets to prevent hemostatic dysfunction in abdominal sepsis.

Clinical management of patients with abdominal sepsis is challenging and largely limited to supportive care. Novel therapeutic options are required to improve clinical outcome of patients with sepsis. It is well established that neutrophil infiltration is a rate-limiting step in septic lung injury^32^. Herein, we observed that depletion of neutrophils not only reduced CLP-induced pulmonary activity of MPO but also decreased lung levels of CXCL1 and plasma levels of CXCL1 and IL-6. Taken together, these findings suggest that neutrophils constitute a key cell in the host response to abdominal sepsis. Septic patients are typified by progressive dysfunction of the coagulation system, including consumption of platelets and coagulation factors causing a state of hypocoagulation^33^. Global tests of hemostasis, such as thromboelastometry and generation of thrombin, have emerged as effective tools to characterize complex coagulopathies^34^. We have recently demonstrated that CLP triggers early TG and that plasma harvested later than 3 hours after induction of CLP exhibits decreased capacity to generate thrombin *ex vivo* due to extensive consumption of coagulation factors^9^. Herein, it was observed that plasma from septic animals had a markedly decreased capacity to generate thrombin, which is in line with four recent clinical reports on patients with sepsis^8,35–37^. Thus, it can be concluded that CLP is a potent inducer of TG. This notion is also supported by our observation that CLP increased plasma levels of TAT complexes. We next examined the role of neutrophils for TG in septic mice and found that neutrophil depletion increased peak and total thrombin formation in plasma from CLP animals, showing for the first time that neutrophils are potent regulators of TG in abdominal sepsis. This notion is also supported by our findings showing that neutrophil depletion reduces plasma levels of TAT in septic animals. In this context, it is also interesting to note that a previous study reported that neutrophil serine proteases can influence fibrin formation and coagulation *in vivo*^23^. Thus, targeting neutrophil activation or function might help to control dysfunctional coagulation in sepsis.

Numerous investigations have reported that NET formation is increased in systemic inflammation and severe infections^38–40^, however, the exact role of NETs in sepsis remains controversial although accumulating evidence suggest that excessive formation of NETs contributes to liver and lung damage in sepsis^40,41^. In the present study, it was found that CLP increased histone 3 citrullination in neutrophils as well as plasma levels of cf-DNA and DNA-histone complexes, which constitute key components of NETs, indicating NET formation in this model, which is in line with previous studies^40,42^. This is also in line with our findings showing co-localization of citrullinated histone 3 and MPO on blood neutrophils from CLP animals. Notably, we found that depletion of neutrophils markedly decreased plasma levels of cf-DNA and DNA-histone complexes in septic animals, suggesting that neutrophils are the likely source of NETs in abdominal sepsis. Administration of DNAse markedly reduced the levels of cf-DNA and DNA-histone complexes in septic animals, showing that DNAse can be used as an effective tool to degrade NET formation *in vivo*. We next asked whether inhibition of NET formation might affect TG in sepsis. It was found that DNAse treatment significantly increased peak and total thrombin formation in plasma from septic animals as well as reduced plasma levels of TAT in septic animals, indicating that NETs indeed regulate TG in sepsis. To further study a direct role of NETs separate experiments on isolated NETs showed that they are potent stimulators of TG. This conclusion is supported by a recent study showing that NETs generated by endotoxin stimulation of neutrophils can induce TG *in vitro*^43^. Considering that thrombin promotes platelet aggregation and that thrombosis aggravates sepsis-associated organ injury^44^, our present findings demonstrating that NETs regulates TG in sepsis reinforce the concept that NETs play a pathological role and promote tissue damage in abdominal sepsis^40^. It is interesting to note that co-incubation with DNAse abolished NET-provoked formation of thrombin, indicating that intact NET structures are critical in triggering TG. In this context, it should be mentioned that PMA-induced NET formation *in vitro* and NET formation *in vivo* might vary in terms of signaling pathways. For example, PMA-induced NET formation is dependent on both NADPH oxidase and MPO whereas many bacterial antigens require NADPH oxidase but not MPO to induce NETs^45,46^. Moreover, some stimuli such as, Staphylococcus aureus and Aspergillus appear to generate NETs independent of NADPH oxidase^45,47^. Herein, we observed that PMA-induced NETs and NETs *in vivo* appear to exert similar type of effects on TG although mechanisms of NET are likely somewhat different.

Activation of neutrophils is not only associated with NET formation but also shedding off cell membrane-derived MPs^24^. Herein, we observed that activated neutrophils increased MP release by more than 6-fold. Considering the observation above that DNAse disinterupts NETs and abolish NET-induced TG, it is interesting to note that treatment with DNAse had no effect on the number of MPs released from activated neutrophils, indicating that intact NETs in combination with MP synergizes in triggering TG. By use of electron microscopy, we observed that NETs were decorated with multiple small rounded particles and immunostaining revealed that these particles avidly bound to annexin V, suggesting that MPs physically interact with NETs. This is the first time in the literature that MPs have been demonstrated to bind to NETs and such NET-MP complexes might have broad implications on various neutrophil-dependent diseases. This concept is also supported by our findings showing that NETs bind to MPs in septic lung injury, suggesting that NET-MP complexes are also found in pathological inflammation *in vivo*. In addition, we found that NET formation in human neutrophils triggered by streptococcal M1 protein also exhibited wide-spread MP binding, indicating that NET-MP complex formation is a more generalizable phenomenon. We addressed the role of these NET-MP aggregates by use of caspase and calpain inhibitors, which have previously been shown to regulate MP formation in platelets^48^. It found that co-incubation of caspase and/or calpain inhibitors abolished generation MPs while NET formation was intact in activated neutrophils, indicating that MP formation in neutrophils is dependent on caspase and calpain signaling. Notably, these NETs depleted of MPs were significantly less effective in inducing TG, suggesting that NETs constitute an assembly scaffold for optimal MP function. In this context, it is interesting to note that we found that inhibition of PAD4 markedly decreased PMA-induced NET formation but had no effect on the density of MPs on the remaining NETs, corroborating that generation of NETs and MPs constitute distinct signaling pathways. Moreover, we observed that co-incubation with wild-type annexin V but not mutated annexin V, which does not bind to PS^28^, abolished MP attachment to NET, suggesting that PS on MP mediates MP-NET interactions. Considering that PS can bind to histones^49^ and that histones constitute nearly 70% of all proteins in NETs^20^, we hypothesized that NET-associated histones could serve as ligands of MP-expressed PS. Indeed, co-incubation with PSA, which binds to and blocks the function of histones^29^, reduced MP binding to NETs by 91%, indicating that histones are the main target of PS on NETs. Taken together, these findings show for the first time that PS-histone interactions mediate MP binding to NETs. This notion is further supported by our findings showing that neutrophil-derived MPs specifically target NETs by binding to histone H4. Although histones are the dominating proteins in NETs^20^, these structures also contain other proteins, including anti-microbial peptides and enzymes, which might also be involved in facilitating MP-NET interactions.

Thrombin is a common end-product of the intrinsic and extrinsic pathways of coagulation. In the present study, we observed that TG triggered by NET-MP complexes was intact in Factor VII-deficient plasma but decreased by more than 97% in Factor XII-deficient plasma. Moreover, it was found that neutrophil-derived MPs avidly bound to Factor XII. Taken together, our results suggest that neutrophil-derived MPs provoke TG predominately via the intrinsic pathway of coagulation. This conclusion is also supported by our observation that a specific inhibitor of Factor XII, CTI, completely blocked NET-induced thrombin formation. These findings raised the question what function NET-MP complex-mediated activation of the intrinsic pathway of coagulation could have in hemostasis. Initial studies suggested that the intrinsic pathway was only of secondary importance due to observations that patients with FXII-deficiency did not suffer from bleeding disorders. In 2005, Renne *et al*. reported that the intrinsic pathway of coagulation facilitates formation and stabilization of three-dimensional thrombi^50^. Knowing that monocytes and monocyte-derived MPs are potent stimulators of the extrinsic pathway in sepsis^51^, it seems plausible that they could induce thrombus formation via the tissue factor-dependent pathway followed by neutrophil-dependent activation of the intrinsic pathway facilitating clot stabilization. Patients with severe sepsis have low levels of intrinsic pathway factors, which is associated with a high mortality risk^35^. Thus, it could be forwarded that the combination of increased monocyte activation and low levels of factors of the intrinsic pathway in patients with sepsis could lead to a state with formation of non-stable clots ending up as microthrombi in the microvasculature. This condition is referred to as disseminated intravascular coagulation and is frequently observed in patients with severe sepsis and constitute a high risk factor for fatal outcome.

We conclude that NET-MP complexes provoke TG via the intrinsic pathway of coagulation in abdominal sepsis. Moreover, our data show that MP binding to NETs is mediated by PS-histone interactions. Taken together, these findings explain complex mechanisms sepsis-induced dysfunction of coagulation and might be useful to target in patients with abdominal sepsis.

## Methods

### Animals

All experiments were conducted in accordance with the legislation on the protection of animals and approved by the Regional Ethical Committee for Animal Experimentation at Lund University, Sweden (permit no. 136–14). Male C57BL/6 mice (20–25 g) were kept in a pathogen free facility on a 12–12 hour light-dark cycle with free access to food and tap water. Mice were housed at least one week before use at a maximum of 7 mice per cage with environment enrichment. Animals were anesthetized with 75 mg of ketamine hydrochloride (Hoffman-La Roche, Basel, Switzerland) and 25 mg of xylazine (Janssen Pharmaceutica, Beerse, Belgium) per kg body weight. The ARRIVE guidelines were consulted for all animal experiments^52^.

### Experimental protocol of sepsis

Abdominal sepsis model was induced by cecal ligation and puncture (CLP) as previously described^53^. Briefly, animals were anesthetized and a midline incision was made to expose the cecum. The cecum was filled with feces from ascending colon, ligated (5–0 silk suture), soaked with PBS and punctured twice with a 21-gauge needle. The cecum was then returned into the peritoneal cavity and the abdominal incision was closed. Animals were treated intraperitoneally (i.p.) with 5 mg/kg DNase (Pulmozyme, Roche, Grenzach-Wyhlen, Germany) or vehicle (PBS) at 1 and 18 h after CLP. To examine the role of PS *in vivo*, animals were treated with recombinant wild-type annexin V and mutant annexin V (M1234-Annexin V, Nexins Research, Hoeven, The Netherlands) i.p. 30 min before CLP. M1234-Annexin V has a single mutation in each of the four calcium binding sites, which abolish the binding affinity to PS^28^. Sham mice underwent the identical laparotomy and resuscitation procedures, but the cecum was neither ligated nor punctured. Samples were collected 24 h after CLP except samples for MPO measurements which were collected 6 h after CLP in re-anesthetized animals.

### Neutrophil depletion

An antibody directed against Ly-6G (20 mg/kg, clone 1A8, Bioxcell, West Lebanon, NH, USA) was given i.p. 24 h prior to CLP induction in order to deplete animals of neutrophils. Control animals received equivalent dose of a control antibody (20 mg/kg, Bioxcell).

### NET quantification *in vivo*

Plasma levels of cf-DNA were measured using a flourogenic assay for doublestranded DNA (Quant-IT PicoGreen dsDNA kit; Invitrogen GmbH). Plasma levels of DNA-histone complexes were quantified by use of a sandwich ELISA based on monoclonal antibodies directed against histones and DNA according to the manufacturer’s instructions (Cell Death detection Elisa plus; Roche Diagnostics).

### NET formation *in vitro*

Freshly isolated bone marrow neutrophils (4 × 10^6^ cells/ml) were incubated with 50 nM PMA (Sigma-Aldrich, Stockholm, Sweden) for 3 h at 37 °C in RPMI 1640. In indicated experiments, cells were co-incubated with PAD4 (200 μM, Cl-amidine, EMD Millipore, Billerica, MA), caspase (50 µM, Z-VAD-FMK, R&D Systems) and/or calpain (25 µM, PD150606, Sigma-Aldrich) inhibitors. Supernatants were discharged and fresh media was added to isolate NETs. Residual neutrophils and NETs were removed through extensive pipetting. The mixture was centrifuged at 200 g (5 min) to remove cellular components and NET-containing supernatants were collected. NET-containing supernatants were further co-incubated with PBS or DNAse for 30 min at 37 °C, and then the media with or without NETs were retrieved for further use *in vitro* experiments.

### TG assay

Blood was collected from the inferior vena cava (1:10 acid citrate dextrose). Platelet poor plasma (PPP) was obtained by centrifugation at 20000 g for 45 min to remove platelets and cell fragments. Human factor VII-, XII-deficient and control PPP (George King Bio-Medical, Overland Park, Overland Park, KS) were kept at -20 °C and centrifuged at 20000 g for 45 min before use. Fluorogenic substrate, Z-Gly-Gly-Arg-AMC, was obtained from Bachem (Bubendorf, Switzerland). A mixture of fluorogenic substrate (0.3 mM) and CaCl_2_ (16 mM) was freshly prepared in Hepes-buffered saline. To trigger the TG *in vitro*, 40 μl PPP, 20 μl fluorogenic substrate with CaCl_2_ and 20 μl samples (Vehicle, NETs) were mixed together with 50 μg/ml DNAse, 50 μg/ml CTI, 100 μg/ml annexin V and 100 μg/ml annexin V M1234 as indicated. TG in animals were studied *ex vivo* in 60 μl of PPP (from Sham or CLP animals) mixed with 20 μl fluorogenic substrate. Formation of thrombin was monitored by measuring accumulated fluorescence using a Tecan Infinite 200 microplate reader (Männedorf, Switzerland) at excitation wavelength 360 nm and emission wavelength 460 nm. All experiments were carried out in duplicate at 37 °C.

### Isolation of bone marrow neutrophils

Mice were euthanized and femurs were collected by careful dislocation of the epiphyses. Bone marrow was flushed from both ends of the bone with a 25-gauge needle and a 2 ml syringe containing Roswell Park Memorial Institute medium 1640 (RPMI 1640, Invitrogen, Stockholm, Sweden) supplemented with 10% fetal bovine serum (FBS, Invitrogen) and 2 mM EDTA (Sigma-Aldrich, Stockholm, Sweden). Following hypotonic lysis (5 ml ice-cold 0.2% NaCl, added for 45 s followed by addition of 5 ml 1.6% NaCl), neutrophils were separated from mononuclear cells by density gradient centrifugation using a Ficoll-Paque gradient (GE Healthcare, Uppsala, Sweden). The neutrophil layer was isolated and washed with RPMI 1640 and cells were resuspended at 4 × 10^6^ cells/ml.

### Flow cytometry

Blood was collected into syringes containing 1:10 acid citrate dextrose and incubated with an anti-CD16/CD32 antibody (10 min at room temperature) to block Fcγ III/II receptors followed by incubation with phycoerythrin-conjugated anti-Ly6G (clone 1A8, BD Pharmingen, San Jose, CA), phycoerythrin-Cy-conjugated anti-Ly6C (clone AL-21, BD Pharmingen), fluorescein isothiocyanatee-conjugated anti-mouse CD3 (clone 145–2C11, BD Pharmingen) and fluorescein isothiocyanatee-conjugated anti-mouse CD19 (clone MB19-1, eBioscience) antibodies. Leukocytes were recovered after centrifugation. NET samples were incubated with FITC-conjugated lactadherin (Haematologic Technologies, Essex Junction, VT) and PerCP Cy5.5-conjugated anti-Gr-1 (clone RB6-8C5, eBioscience, Solna, Sweden) antibodies. After labeling, 40 μl calibration beads with a size of 1 μm (Flow Count, Beckman Coulter, Brea, CA) were added and the mixtures were diluted to 300 μl with 0.2 μm filtered PBS. Flow cytometric analysis was performed according to standard settings on a FACSCalibur flow cytometer (Becton Dickinson, Mountain View, CA), and a viable gate was used to exclude dead and fragmented cells. Neutrophils were defined as Ly6C+/Ly6G+ cells and MP were defined as Gr-1+/lactadherin+ particles with a size less than 1 μm. Flow cytometric detection of NETs were performed as previously described^54^. In brief, red blood cells were lysed with ammonium-chloride-potassium lysis buffer, fixed in 2% paraformaldehyde and blocked with 2% bovine serum albumin (30 min). Cells were then incubated with an anti-CD16/CD32 antibody to block Fcγ III/II receptors followed by incubation with the primary anti-histone H3 antibody (citrulline 2,8,17, ab5103; Abcam, Cambridge, MA), allophycocyanin-conjugated secondary antibody (A-21038, Thermo Scientific, Rockford, IL), FITC-conjugated anti-MPO antibody (mouse: ab90812) and phycoerythrin-conjugated anti-Ly6G antibody.

### MPO activity

Lung tissue samples were harvested 6 h after induction of CLP, frozen immediately and then thawed, weighed and homogenized in 0.02 M PB (pH 7.4). After centrifugation for 10 min at 19000 g at room temperature, pellets were dissolved in 1 ml of 0.5% hexadecyltrimethylammonium bromide. Samples were frozen and the thawed in a water bath (2 h at 60 °C) and then sonicated (90 s). After 5 min of centrifugation at 19000 g, supernatants were collected to determine the MPO activity by spectrophotometry. The MPO-catalyzed redox reaction of H_2_O_2_ result in the change of absorbance (450 nm, with a reference wavelength 540 nm, 25 °C). Values were expressed as MPO units per g tissue.

### Enzyme-linked immunosorbent assay (ELISA)

Plasma and lung levels of IL-6, CXCL1 (R&D Systems, Abingdon, UK, Siemens, Marburg, Germany) and thrombin-anti-thrombin complex (TAT, Siemens, Marburg, Germany) were determined by commercial ELISA kits. Lung tissue was harvested 24 h after induction of CLP and immediately frozen in liquid nitrogen. Blood samples were collected 6 or 24 h after induction of CLP from the vena cava (1:10 acid citrate dextrose) and centrifuged at 19000 g for 10 min at 4 °C and stored at -20 °C until use. Linearity was assessed and confirmed by serial dilution of standards containing recombinant mouse IL-6, CXCL1 and TAT and a calibrator diluent.

### Surface plasmon resonance

Analyses were performed with a BIAcore X100 instrument (GE Healthcare, Uppsala, Sweden) using Sensor Chip CM5 technology at 25 °C in a HBS-EP buffer (10 mM HEPES, 150 mM NaCl, 3 mM EDTA, 0.05% (v/v) Surfactant P20, pH 7.4). Histone H4 (Abcam, Cambridge, UK) was diluted in sodium acetate (10 mM, pH 5.5) and immobilized via amine coupling to flow cell 2. Flow cell 1 was subjected to the coupling reaction but without protein, and was used as a control in each experiment. MP at indicated concentrations was injected over the coated surface at 30 μl/min in running buffer. Regeneration of sensorchip surfaces was obtained by injection of 30 μl 50 mM NaOH.

### Transmission electron microscope and scanning electron microscope

Deparaffinized lung tissue samples and neutrophils stimulated with PMA as described above were fixed in 2.5% glutaraldehyde in 0.15 mol/L sodium cacodylate, pH7.4 (cacodylate buffer), for 30 min at room temperature. Specimens were washed with cacodylate buffer and dehydrated with an ascending ethanol series from 50%(vol/vol) to absolute ethanol (10 min/step). The specimens then were subjected to critical-point drying in carbon dioxide, with absolute ethanol as intermediate solvent, mounted on aluminum holders, and finally sputtered with 20 nm palladium/gold. Specimens were examined in a Jeol/FEI XL 30 FEG scanning electron microscope at the Core Facility for Integrated Microscopy at Panum Institute (University of Copenhagen, Denmark). The location of individual target molecules was analyzed at high resolution by ultrathin sectioning and transmission immunoelectron microscopy. Specimens on coverslips were embedded in Epon 812 and sectioned into 50-nm–thick ultrathin sections with a diamond knife in an ultramicrotome. For immunohistochemistry, sections were incubated overnight at 4 °C with primary antibodies against elastase, CD41, Mac-1, histone H4, DNA and Factor XII (Abcam, Cambridge, UK). Controls without primary antibodies were included. The grids then were incubated with species-specific, gold-conjugated secondary antibodies (Electron Microscopy Sciences, Fort Washington, MD). Gold-labeled annexin V were also used. Finally, the sections were postfixed in 2% glutaraldehyde and post-stained with 2% uranyl acetate and lead citrate. Specimens were observed in a Jeol/FEI CM100 transmission electron microscope operated at 80-kV accelerating voltage at the Core Facility for Integrated Microscopy at Panum Institute.

### Human neutrophils

Neutrophils were purified from blood of healthy donors using polymorphprep (Axis-Shield, Oslo, Norway) as previously described^55^. Cells were washed and diluted in ME-Medium (Invitrogen) with HEPES buffer. 50 μl 3 × 10^6^ cell per ml were seeded on polylysine-coated slides and activated with M1 protein (0.5 μg/ml) for 2 h. Specimens were fixed at room temperature (overnight) with 2% formalin followed by a post fixation step with 2.5% glutaraldehyde in 150 mM cacodylate buffer at room temperature (overnight) as previously described^56^. Specimens were examined in a DELPHI correlative light and electron microscope (Phenom-World, Eindhoven, Netherlands). The use of cells purified from human blood was approved by the Ethics Committee at Lund University, Lund, Sweden (permit no. 2015/801). Written informed consent was obtained from all healthy donors or their legal guardians. All methods involving human blood samples were performed in accordance with the relevant guidelines and regulations.

### Western blot analysis

Citrullinated histone 3 protein was determined in neutrophils isolated from the blood of mice 24 h after CLP induction. Proteins (20 μg per lane) were separated by sodium dodecyl sulphate-polyacrylamide gel electrophoresis on 15% polyacrylamide gels and transferred onto nitrocellulose membranes (Bio-Rad Laboratories, Hercules, CA). Membranes were blocked in 0.05% PBS-Tween containing 5% milk (Bio-Rad Laboratories) and incubated with the anti-histone H3 antibody (citrulline R2 + R8 + R17, 1:1,000, Abcam) at 4 °C overnight. The primary antibody was detected by incubation with horseradish peroxidase-coupled second antibody (1:3,000 in PBS-Tween with 5% milk) at room temperature for 1 h. Chemiluminescence was detected by using Western Lighting Chemiluminescence Reagent Plus (PerkinElmer LAS, Inc., Boston, MA). Films were developed using a standard photographic procedure and quantitative analysis of detected bands was carried out by densitometer scanning using VersaDoc Imaging System (BioRad Laboratories). Total protein was used as a loading control.

### Statistics

Data are presented as mean values ± standard error of the mean (SEM). Statistical evaluations were performed using Kruskal-Wallis one-way ANOVA on ranks, followed by multiple comparisons (Dunnett’s method). *P* < 0.05 was considered statistically significant and *n* represents the number of animals.

## Electronic supplementary material

Supplemental Information
