# Supplementary material for: Neutrophil extracellular trap-microparticle complexes enhance thrombin generation via the intrinsic pathway of coagulation in mice
Source: Sci Rep. 2018 Mar 5;8:4020. doi: 10.1038/s41598-018-22156-5 (PMC5838234; doi:10.1038/s41598-018-22156-5)

Neutrophil extracellular trap-microparticle complexes enhance thrombin generation via the intrinsic pathway of coagulation in mice

Yongzhi Wang [1,a], Lingtao Luo [1,4,a], Oscar Ö Braun [2], Johannes Westman [3,5], Raed Madhi[1], Heiko Herwald [3], Matthias Mörgelin [3], and Henrik Thorlacius [1,b]

[1]Department of Clinical Sciences, Malmö, Section for Surgery, [2]Department of Clinical Sciences, Lund, Section of Cardiology, [3]Department of Clinical Sciences, Lund, Division of Infection Medicine, Lund University, Sweden, [4]Department of Surgery, The First Affiliated Hospital of Xiamen University, Xiamen, China, [5]Program in Cell Biology, The Hospital for Sick Children, Toronto, Canada.

---

[a]These authors contributed equally

[b]**Correspondence to:**

Henrik Thorlacius, MD, PhD

Department of Clinical Sciences, Malmö

Section for Surgery, Lund University

205 02 Malmö, SWEDEN

Telephone: Int+46-40-331000

Telefax: Int+46-40-336207

E-mail: henrik.thorlacius@med.lu.se

Supplemental Figures and Figure legends

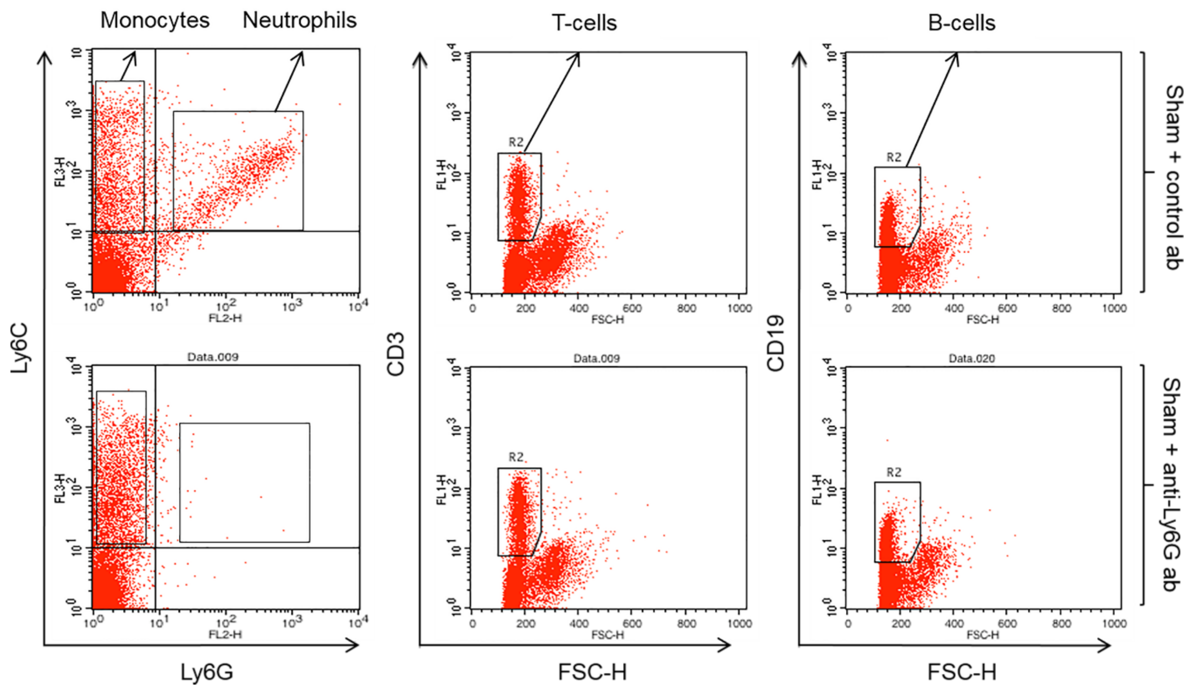

Supplemental Figure 1. Neutrophil depletion. Mice underwent CLP or the identical laparotomy and resuscitation procedures, but the cecum was neither ligated nor punctured (Sham). Animals received intraperitoneal injections of the anti-Ly-6G antibody (Anti-Ly-6G ab, 20 mg/kg) or a control antibody (Ctrl ab, 20 mg/kg) prior of CLP induction. Flow cytometry was used to identify neutrophils (Ly6G^{+}/Ly6C^{+} cells), monocytes (Ly6G^{-}/Ly6C^{+} cells, T-cells (CD3^{+} cells) and B-cells (CD19^{+} cells) depicted in dot plots.

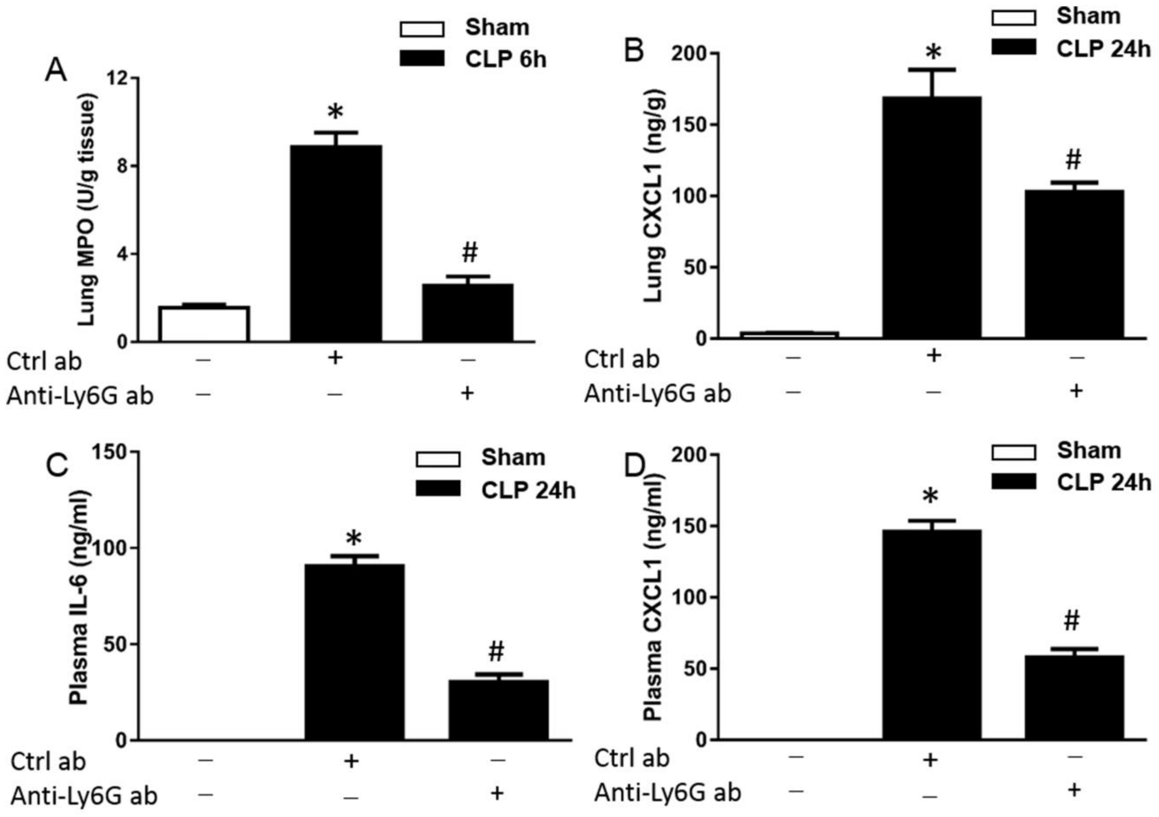

Supplemental Figure 2. Neutrophils regulate systemic inflammation and TG in abdominal sepsis. Levels of A) MPO and B) CXCL1 in the lung as well as plasma levels of C) IL-6 and (D) CXCL1 were determined at indicated time-points after CLP induction. Data are presented as mean ± SEM and $n = 5$. *$P < 0.05$ *vs*. Sham and $^{\#}P < 0.05$ *vs*. Ctrl ab + CLP.

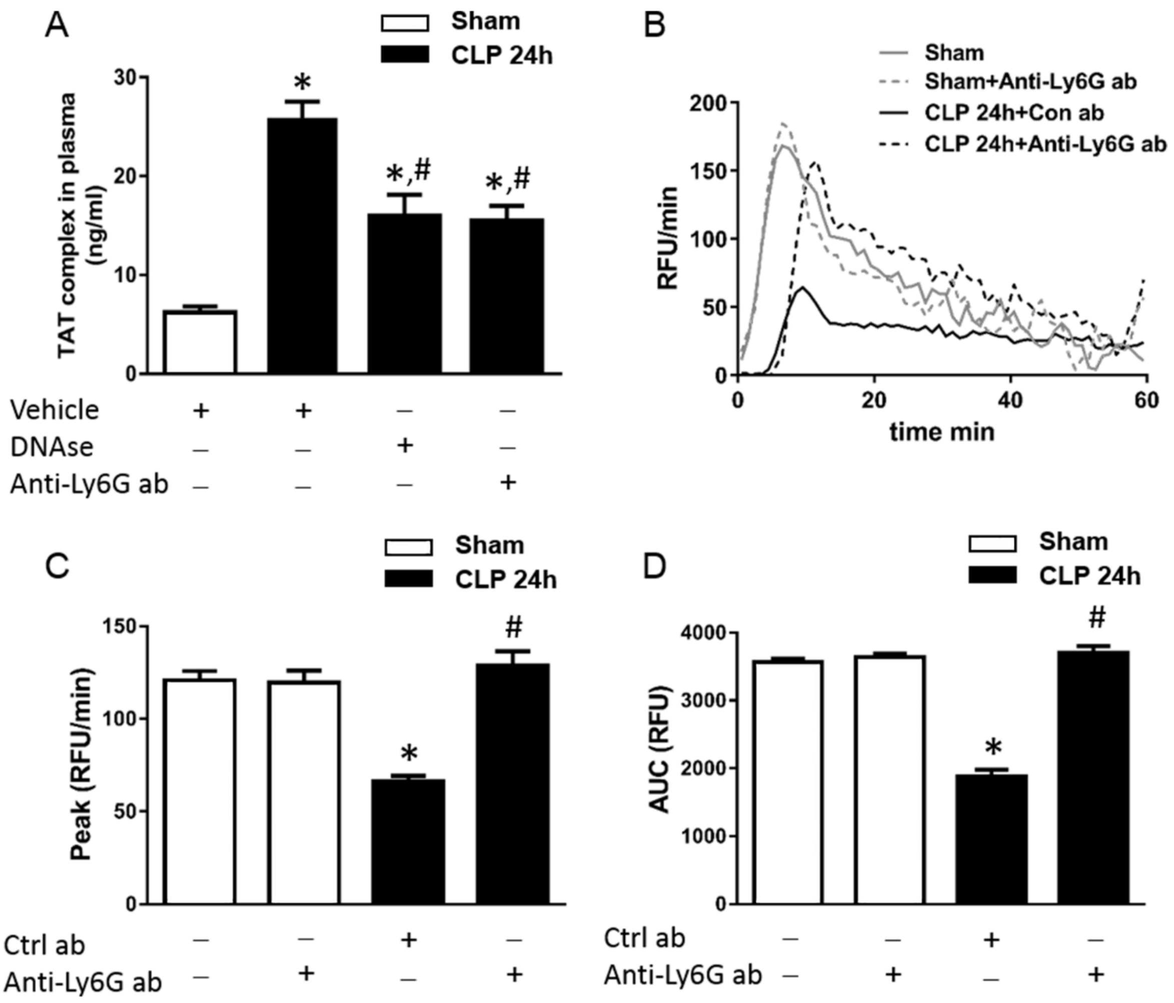

Supplemental Figure 3. Thrombin generation. (A) CLP increases plasma levels of TAT complexes. Plasma levels of TAT complexes were determined by use of ELISA. (B) TG over time, (C) peak and (D) total levels of TG were determined as described in Materials and Methods. Mice underwent CLP or the identical laparotomy and resuscitation procedures, but the cecum was neither ligated nor punctured (Sham). Animals received intraperitoneal injections of the anti-Ly-6G antibody (Anti-Ly-6G ab, 20 mg/kg) or a control antibody (Ctrl ab, 20 mg/kg). Data are presented as mean ± SEM and $n = 5$. *$P < 0.05$ *vs*. Sham and $^{\#}P < 0.05$ *vs*. Ctrl ab + CLP.

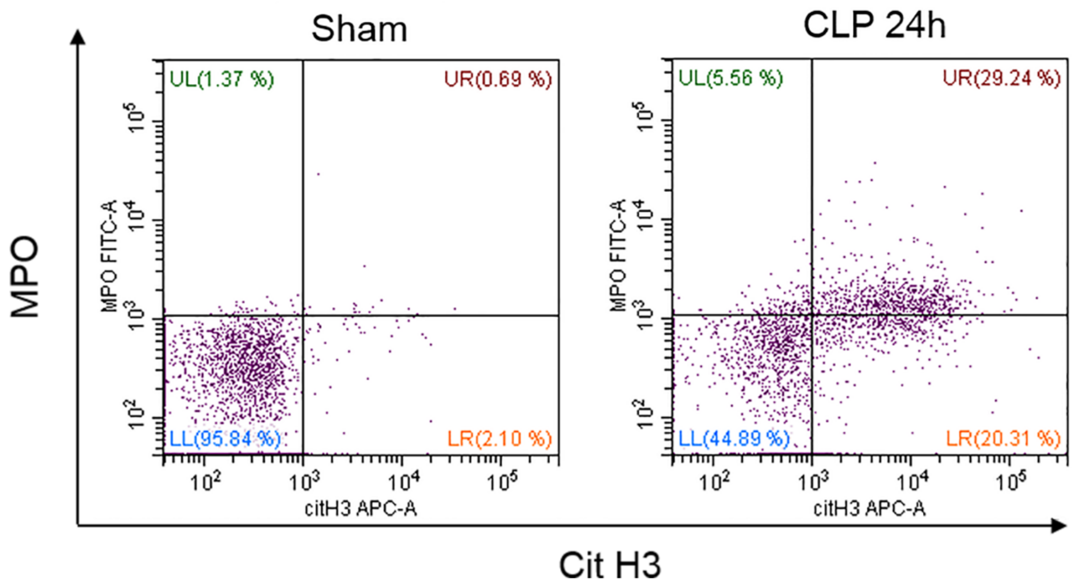

Supplemental Figure 4. Flow cytometric detection of NETs. Blood was obtained from sham and CLP 24h animals. Red blood cells were lysed and fixed, then cells were incubated with a primary anti-histone H3 antibody (citrulline 2,8,17), allophycocyanin-conjugated secondary antibody, FITC-conjugated anti-MPO antibody and phycoerythrin-conjugated anti-Ly6G antibody. Representative flow data showing co-localization of citrullinated histone 3 and MPO on neutrophils (Ly6G+) from the blood of CLP mice.

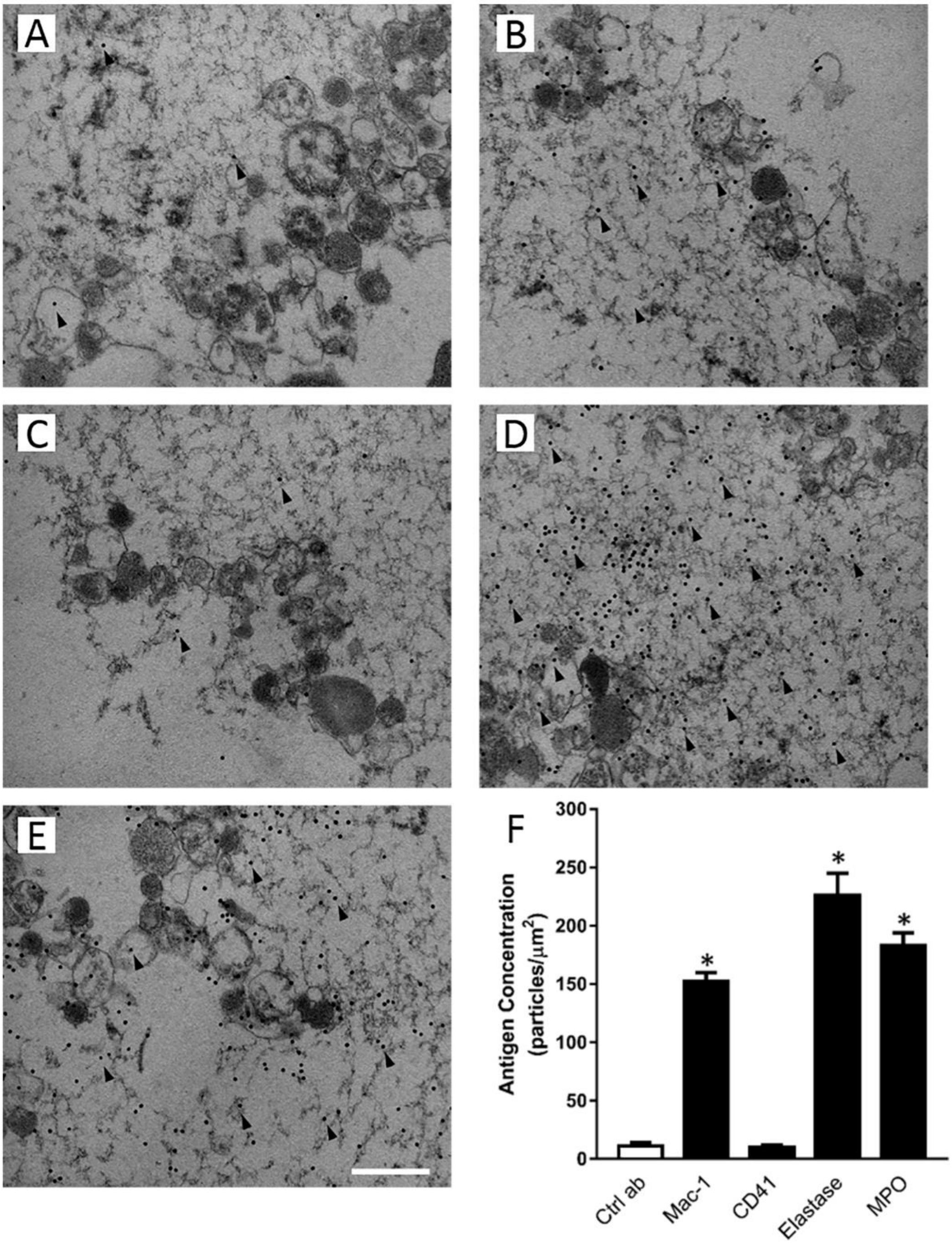

Supplemental Figure 5. NETs were generated by PMA-stimulation of bone marrow neutrophils. Transmission electron microscopy showing NETs incubated with a gold-labeled (A) control antibody, (B) anti-Mac-1 antibody, (C) anti-CD41, (D) anti-elastase antibody and E) anti-MPO antibody. Selected antigens are indicated by black arrowheads. Scale bar = 0.25 µm. (F) Antigen concentration on NETs. Data are presented as mean ± SEM and $n = 5$. *$P < 0.05$ *vs*. Ctrl ab.

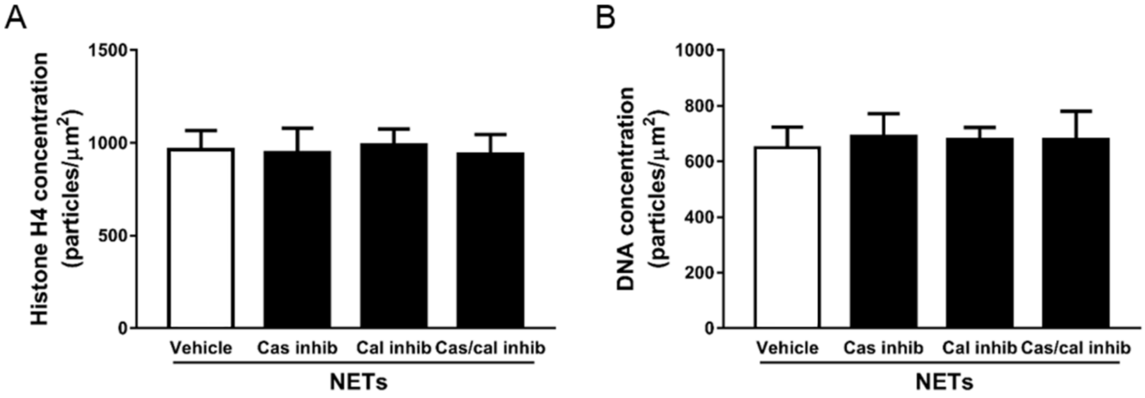

Supplemental Figure 6. DNA and histone contents in NETs. NETs were generated from PMA-stimulated bone marrow neutrophils co-incubated with vehicle, caspase inhibitor, calpain inhibitor or a combination of caspase and calpain inhibitors. NETs incubated with gold-labeled anti-DNA or anti-histone H4 antibodies. Transmission electron microscopy was used to calculate the density of (A) DNA and (B) histone H4 in the NETs. Data are presented as mean ± SEM and *n* = 5.

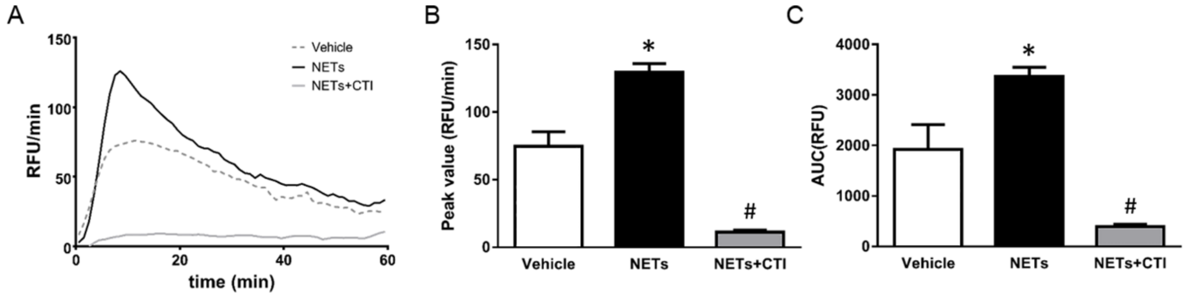

Supplemental Figure 7. NET-induced generation of thrombin is mediated via the intrinsic pathway of coagulation. NETs were generated from PMA-stimulated bone marrow neutrophils. Platelet poor plasma was challenged with NETs and vehicle or corn trypsin inhibitor (CTI). (A) TG over time, (B) peak and (C) total levels of TG were determined as described in Materials and Methods. Data are presented as mean ± SEM and n = 5. *P < 0.05 *vs*. Vehicle and #P < 0.05 *vs*. NETs.

Full-length gels and blots

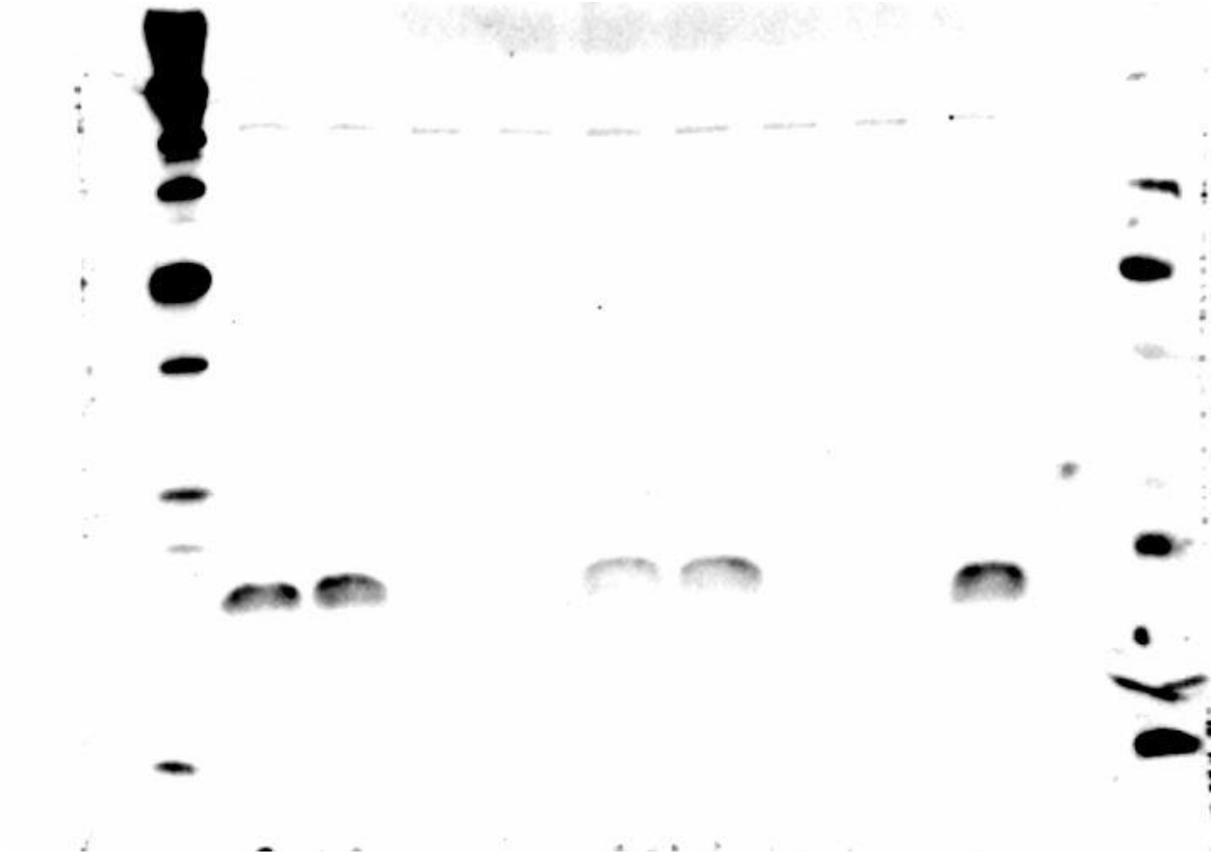

Supplement: Supplementary file 1 — Supplemental Information [file 41598_2018_22156_MOESM1_ESM.pdf]
